# 

**Published:** 1888-09-29

**Authors:** 


### CONTENTS.

**Voluntary Chaplains** - - - - - 413

**Portents and Epidemics** - - - - - 414

**The Doctor's Pulpit**.—Mental Depression - - - 415

**Hints for the Sick-room.** By M. M. BASIL, M.A., M.B.—Administration of Medicines - - - - - 416

**Physiology and its Uses.**—The Bones of the Leg - - 417

**New Remedies and Appliances** - - - - 418

**The Editor's Letter-box** - - - - - 418

**Voyages in Vacation.** I.—By ONE IN SEARCH OF RESTFUL RESTORATIVES - - - - - - - - 419

**Notes and News** - - - - - - - 420

**Everybody's Page** - - - - - - - 422

**Annotations.**—Brag or Benevolence?—A "Sweated" Lady.—Committee-men and Business Contracts - - - - 423

**Turning Over New Leaves.**—The Intestinal Diseases of Childhood.—Canal Boats and Boatmen - - - - 424

**An Ishmaelite.** By LESLIE KEITH, Author of "The Chilcotes," "East and West," etc. - - - - - - 425

**A Great Scottish Hospital** - - - - - 426

**The Children's Corner** - - - - - - 428

**EXTRA SUPPLEMENT.—THE NURSING MIRROR.**

En Passant.—A Lesson in Physiology.—The Fatal Mistake at Belfast.—The Nursing of Ovariotomy.—A True Story.—Another True Story - - - - - - - - ci
Everybody's Opinion.—A Nurse's Position.—Nursing Ovariotomy Cases.—The Question of Courage.—Volunteer Nurses - - cii
Letters from a Probationer.—IV. - - - - - - cii
Lectures - - - - - - - - ciii
Appointments - - - - - - - ciii
Vacancies - - - - - - - - ciii
Notes and Queries - - - - - - - ciii

